# A high-content image analysis approach for quantitative measurements of chemosensitivity in patient-derived tumor microtissues

**DOI:** 10.1038/s41598-017-06544-x

**Published:** 2017-07-26

**Authors:** Ilmari Ahonen, Malin Åkerfelt, Mervi Toriseva, Eva Oswald, Julia Schüler, Matthias Nees

**Affiliations:** 1 Department of Mathematics and Statistics, University of Turku, Turku, Finland; 2 Institute of Biomedicine, University of Turku, Turku, Finland; 3 Discovery Services, Charles River, Freiburg, Germany

## Abstract

Organotypic, three-dimensional (3D) cancer models have enabled investigations of complex microtissues in increasingly realistic conditions. However, a drawback of these advanced models remains the poor biological relevance of cancer cell lines, while higher clinical significance would be obtainable with patient-derived cell cultures. Here, we describe the generation and data analysis of 3D microtissue models from patient-derived xenografts (PDX) of non-small cell lung carcinoma (NSCLC). Standard of care anti-cancer drugs were applied and the altered multicellular morphologies were captured by confocal microscopy, followed by automated image analyses to quantitatively measure phenotypic features for high-content chemosensitivity tests. The obtained image data were thresholded using a local entropy filter after which the image foreground was split into local regions, for a supervised classification into tumor or fibroblast cell types. Robust statistical methods were applied to evaluate treatment effects on growth and morphology. Both novel and existing computational approaches were compared at each step, while prioritizing high experimental throughput. Docetaxel was found to be the most effective drug that blocked both tumor growth and invasion. These effects were also validated in PDX tumors *in vivo*. Our research opens new avenues for high-content drug screening based on patient-derived cell cultures, and for personalized chemosensitivity testing.

## Introduction

Despite some recent therapeutic advances, lung cancers remain a leading cause of cancer-related deaths. In particular, late stage, aggressive non-small cell lung carcinoma (NSCLC) is a leading cause of cancer-related mortalities worldwide^1^. NSCLCs likely derive from epithelial cells of the proximal airway (squamous cell carcinomas) or from distal alveolar locations (adenocarcinomas). The development of novel personalized therapeutic strategies, the introduction of more predictive biomarkers including EGFR^2^ and successful application of novel tyrosine kinase inhibitors that target for example the ALK oncogene^3^, have improved patient response to therapies. However, the pronounced heterogeneity of most NSCLCs, coupled with high genetic instability contributes to the development of drug resistance, resulting in progression and failure of therapies. Invasion, metastasis and recurrence are thus the main consequences leading to poor patient outcome. The major cause of death from NSCLC remains to be due to local and distant metastases.

The availability of validated lung cancer models that resemble the heterogeneous histology of solid human tumors is still a significant bottleneck for preclinical cancer research and drug discovery. It becomes increasingly apparent that both established tumor cell lines, and standard mouse models based on such cell lines (subcutaneous xenografts), only poorly recapitulate the morphology and heterogeneity seen in clinical lung cancer biopsies. Genetically engineered mouse models partly fill the gap, but they remain expensive and provide very low experimental throughput. Furthermore, tumors derived from other species and different genomes often deviate in behavior from human cancers. As a consequence, patient-derived xenografts (PDXs), derived from surgically resected NSCLC tumors, increasingly become established as biologically and clinically more relevant model systems^4, 5^. Recent results provide strong evidence that PDXs established from lung cancers indeed closely mimic the characteristics of patient primary tumors^6, 7^. The presence of integral murine stroma in PDX models, which rapidly replace the human counterparts after engraftment, is not necessarily a limitation for functional and chemosensitivity studies^8^.

Three-dimensional (3D) cell culture models of epithelial cancers, increasingly utilized for high-content drug screening methodologies, have allowed researchers to gain insight into the biological and cellular mechanisms of the disease^9–11^. During recent years, the technology and practices related to *in vitro* 3D cell models have greatly advanced, and now represent more and more realistic solutions for disease modeling. Major technological advances in organotypic, 3D models or microtissues have enabled the investigation of complex structures. These mimic the histologies observed in different solid cancers in a much more realistic fashion^12^, specifically also in lung cancers^13^. A major drawback even of advanced 3D models remains the uncertain biological relevance of the “classic” cancer cell lines that are still routinely utilized. Regardless of the level of technical sophistication, 3D models based on cell lines that have been cultured for decades on plastic dishes cannot fully address the complexity and heterogeneity, nor the genetic instabilities observed in patients’ tumors. Genetic profiling of cell lines has shown the occurrence of large numbers of mutations probably irrelevant for the disease, as most of these were likely acquired *in vitro*. Similarities between cell lines are often more pronounced than their resemblance to the original tumor entity they were derived from^14, 15^. Clinically truly relevant and personalized *in vitro* disease models can only be achieved by utilizing patient-derived primary cell material. Small quantity of starting material is often a limitation for isolation of patient tumor cells directly from biopsies. However, the tumor mass can be increased as PDX cultures from which subsequently a larger number of tumor cells can be isolated for culture *in vitro*. Such PDX cultures represent natural co-cultures of human cancer and mouse fibroblast cells, which form complex epithelial tissue-like structures (organoids), surrounded by mesenchymal murine fibroblasts (the tumor stroma), when embedded in extracellular matrix (ECM). We have previously demonstrated that functional tumor-stroma-interactions and the tumor microenvironment (TME) are important to faithfully mimic tumor-specific drug responses *in vitro* ^16^. In addition, we and others showed that cancer-associated fibroblasts (CAFs) demonstrate altered, activated functions compared to normal fibroblasts^17^. Thus, CAFs have a significant impact on both tissue formation and drug sensitivity. Tumor-stroma interactions occur by the indirect cross talk via chemokines and growth factors as well as by direct cell-cell interactions. Mouse fibroblasts in PDX tumors appear to fully replace human fibroblast functions and thus compensate for their loss in both PDX and functional *in-vitro* microtissues.

A number of relatively straightforward image analysis methods, mainly for comparably simple 3D mono-cultures, have been described in the literature, and a collection of both general and dedicated image analysis software tools is now available for the analysis of 3D spheroid cultures. BisQue^18^, CellProfiler^19^, Icy^20^ and ImageJ^21^ are popular open-source software, all offering a range of functionalities for a variety of image analysis problems. Instead of taking a more focused approach to a particular problem, these software applications generally sacrifice some of the user-friendliness for the sake of increased customizability. Also the open-source software BioimageXD^22^ maintains a high repertoire of readily available tools, but is not ready for fast analysis of large image sets. Another solution for high-throughput image analysis, AMIDA, has been developed in our laboratory^23, 24^. Despite a wide array of morphometric readout options available in AMIDA, the currently available functions do not yet match the detailed requirements for complex primary co-cultures, or PDX cultures. An advanced segmentation method suitable for complex 3D co-cultures of cancer cells with CAFs, based on Markov random fields, was introduced by Robinson *et al*.^25^. In addition, we have demonstrated that the complex, heterogeneous and dynamic interaction of tumor and stromal compartments can be simultaneously investigated by tracking their growth, morphogenesis and movement over time and space^16^. Another 3D co-culture setup of human prostatic tissues was presented, where their behavior was assessed through systematic measurements of morphology and mobility^26^. These findings and recent contributions by others^27, 28^, argue for the biological relevance of 3D tumor-fibroblast co-cultures in cancer research and personalized medicine, and highlight the need for further development both in the laboratory and in the analysis methodology.

Here, we have established PDX microtissue cultures derived from NSCLC patients. The cultures were treated with standard of care drugs in order to study their drug-sensitivity. We have developed a pipeline of analyses suitable for the PDX microtissue image data, including the early image processing and the subsequent statistical analysis of specific treatment effects. A local entropy filter and a global thresholding method were used to separate the tissues from the background in the original image data. This foreground was then automatically classified into either tumor or fibroblast cells, based on a set of texture features. The overall growth of each cell type, the area and roundness of the multicellular organoid structures were then quantified using appropriate morphological features. Representative drug sensitivity tests on PDX microtissues *in vitro* were performed, leading to markedly different distributions of morphologies. These results demonstrate the potential of the analysis methods to quantify individual drug responses of distinct patient samples. Thus, our results provide a promising methodology useful for high-content analysis of complex patient-derived cancer cultures.

## Results

### Establishment of patient-derived microtissue models

**Figure 1:**
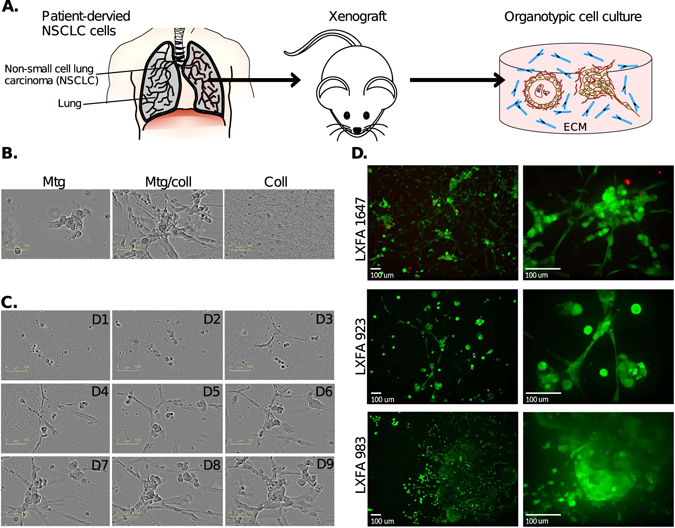
Patient-derived xenograft (PDX) microtissue models of non-small cell lung carcinoma (NSCLC). (**A**) Cells from NSCLC patients were isolated and propagated as mouse xenografts to maintain and enrich for the tumor material. Tumor and stromal cells were isolated as single cell suspensions, a fraction of which was directly cultured for 12 days as organotypic cultures embedded in extracellular matrix (ECM). (**B**) Optimization of extracellular matrix (ECM) preparations used for 3D culture, using Matrigel, type I collagen, and a 1:1 mixture of both. (**C**) Spontaneous PDX microtissue generation (LXFA 1647) from single cells, for 9 days of culture in ECM (Matrigel-collagen). (**D**) Live-cell staining for living cells with Calcein AM (green). Confocal imaging of representative PDX microtissues shows characteristic morphologies formed by PDX tumors LXFA 923, LXFA 983, and LXFA 1647. A blow up image is shown on the right. Scale bars: 100 *μm*. The first two images in A are distributed on *pixabay*.*com* under CC0 Public Domain Licence.

In order to develop cell culture models that resemble cancer tissues, and more reliably recapitulate the complex architecture of solid tumors *in vivo*, PDX cell culture models were established *in vitro* (Fig. 1A). NSCLC tumors from patients were cut into pieces, grown as PDX tumors in mice, and cell suspensions for these tumors were directly transferred to organotypic 3D cultures. This approach completely circumvents standard two-dimensional (2D) culture on plastic plates, which can dramatically change the characteristics of the tumor cells^14^. In this study, three NSCLC patient samples (LFXA 923, LFXA 983, LFXA 1647) were inoculated to generate tumors in mice. The resulting PDX tumors were extracted, DNA was isolated, and sequenced for characterization of mutations in key regulatory proteins and drivers of NSCLC. The mutation hot spots investigated covered the most frequently mutated oncogenes and tumor suppressor genes in lung cancer, including TP53, KRAS, EGFR, MET and LKB1 (Suppl. Table 1). LFXA 983 and LFXA 1647 tumors displayed a homozygous deletion of TP53, whereas LXFA 923 showed a homozygous deletion of the LKB1 gene. LFXA 923 and LFXA 983 both displayed heterozygous deletion of KRAS. The mutation analyses clearly demonstrated individual differences in tumor genotypes.

Cell suspensions were generated from the PDX tumors, which consisted invariably of a mixture of mouse fibroblasts with human NSCLC cells. This model allows the investigation of the growth of multicellular tumor organoids *in vitro* and simultaneously monitor the proliferation of fibroblasts. 3D culture of cell suspensions formed natural *in vitro* co-cultures between tumor and stroma when embedded into biologically relevant ECM preparations. Under organotypic cell- and tissue culture conditions, NSCLC tumor cells either formed tight multicellular structures (organoids) or lose aggregates with variable degrees of cell-cell interactions.

To generate representative, robust and reproducible PDX microtissue-cultures based on NSCLC patient samples, we adapted a standardized and miniaturized protocol^16, 23^. According to real-time cell monitoring, almost every single tumor cell seeded into ECM gave rise to a multicellular tumor organoid with clonal origin. To guarantee this strictly clonal approach, single cell suspensions were meticulously tested not to contain any cell clusters. Furthermore, cell seeding density was restricted (300–700 cells/well) in order to avoid early fusion of emerging organoids as the result of close-by neighbors. Hundreds of isolated, independent organoids spontaneously formed per well. To find the most suitable ECM composition to support the growth of both, tumor cells and stromal fibroblasts, three types of ECM were utilized: Matrigel, rat tail type I collagen, and a mixture of these (1:1). In accordance with our previous findings^16^, in collagen, the fibroblasts displayed elongated and branching morphology which formed an extensive network, but remained only small and round in Matrigel (Fig. 1B). A mixture of both Matrigel and collagen provided a suitable compromise that supports the growth of both epithelial tumor cells and mesenchymal fibroblasts.

Further microtissue model development focused on the 1:1 mixture of Matrigel and collagen, which best recapitulates the formation of characteristic *in vivo* morphologies of both compartments of solid tumors. The growth and spread of the cancer and fibroblast cells in ECM was further investigated by real-time monitoring, using the IncuCyteTM live-cell imaging system (Fig. 1C), which relies on phase contrast microscopy. Functional microtissues started to emerge after 2–3 days, and larger organoids or loose clusters of tumor cells were generated over 2–5 days. These were surrounded by a dense meshwork of fibroblasts, many of which were in direct physical contact to the tumor aggregates at multiple positions. At day 10 of 3D culture, cultures were stained with Calcein AM live cell dye and high-resolution image stacks of living PDX co-cultures were acquired by spinning-disc confocal microscopy. Calcein AM staining revealed the formation of multiple, multicellular organoids with clear morphological differences between the three PDX cultures (Fig. 1D). Many loose and grape-like structures were observed that indicate tumor aggregates or clusters with limited cell-cell-contacts; characteristic of aggressive tumors like advanced lung cancers. LXFA 983 contained many fibroblasts, which caused severe contraction of the 3D culture well after day 8–10. In contrast, both LXFA 923 and LXFA 1647 tumor suspensions, which contained less fibroblasts, generated stable and reproducible microtissues. At day 8–10, these cultures were sufficiently mature for drug target validation and phenotypic screening. The differences in the genotype and mutation spectra between the three different patient cells (Suppl. Table 1) offer possible justifications to the variations in the observed phenotype.

**Figure 2:**
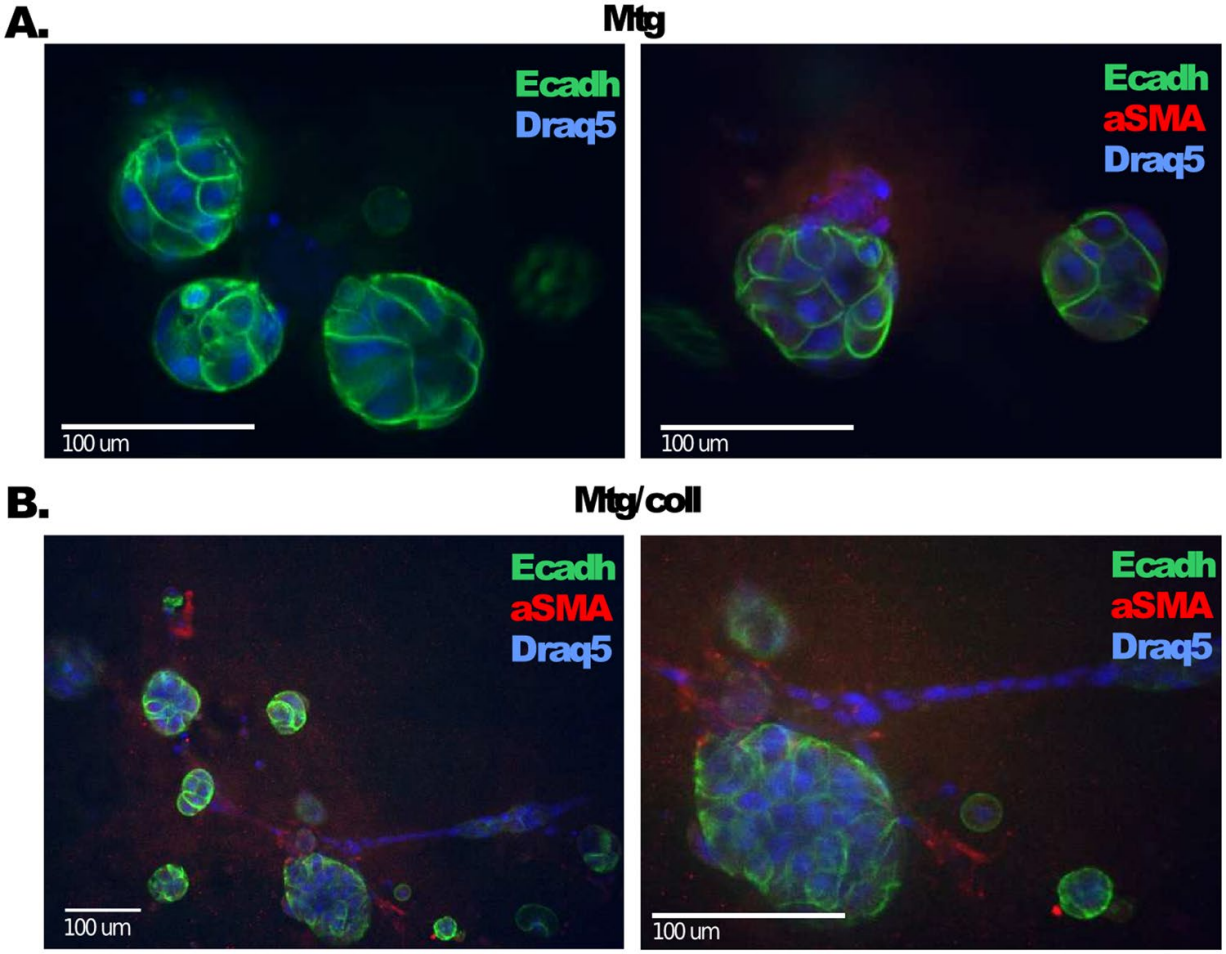
Characterization of PDX microtissues using immunofluorescence. The fibroblasts-tumor interaction was characterized in different ECM preparations. (**A**) Matrigel and (**B**) in Matrigel/collagen mixture. Fibroblasts require collagen for growth and spreading. Roundish tumor organoids express epithelial markers such as E-cadherin (Ecadh; green), whereas the fibroblasts express mesenchymal markers such as alpha-Smooth Muscle Actin (aSMA; red). Nuclei are stained with Draq5 (Blue). Scale bars: 100 *μm*.

To establish accurate computational methods for the analysis of confocal images of the PDX microtissues, the two cell populations first needed to be reliably identified and distinguished from each other. Immunofluorescence stainings with monoclonal antibodies targeting selected mouse and human proteins specific for each cell type were performed. In Matrigel, the roundish multi-cellular structures typically expressed human E-cadherin, a marker for epithelial cells (Fig. 2A). Large and small cancer expressing smooth muscle actin (*α*SMA), a mesenchymal marker often expressed by fibroblasts. In contrast, in Matrigel/collagen mixture the *α*SMA expressing fibroblasts rapidly spread and formed characteristic branches, whereas the tumor cells generated multicellular tumor organoids (Fig. 2B).

### Generation of image data on diverse PDX microtissue cultures for method evaluation

To evaluate the image analysis method, we generated a low resolution confocal image data set of PDX microtissue cultures. This data set was of exploratory nature, containing images from all three NSCLC patient cells (LXFA 923, LXFA 983 and LXFA 1647) and from three distinct culture conditions: 1) “Freezer”: the PDX cell suspensions were immediately propagated after thawing, and directly placed into ECM to circumvent even short-term 2D cultures. 2) “Pre-cultured”: these cells were also thawed but first cultured for three days in a 10 cm petri dish. Here, a large portion of fibroblasts were able to attach the plastic surface. This approach positively selected for tumor cells that remained in the non-adherent fraction. 3) “Cell line”: the mouse fibroblast “contamination” was completely removed by magnetic cell sorting. These isolated, exclusively human tumor cells were then cultured for a limited number of passages in standard 2D monolayer culture, prior to their transfer into organotypic cell culture. Using these three different pre-conditions prior to seeding into 3D cultures, the number of fibroblasts and the ratio of fibroblasts to tumor cells originally present in the PDX suspensions were effectively diminished and could be strictly controlled.

Live cell confocal images of Calcein-stained PDX cultures were obtained at day 12 of 3D culture. The final image data sets were highly diverse and captured a broad view of the spectrum of possible morphologies that form in organotypic 3D cultures. These image series formed the solid basis for optimizing appropriate and effective image analysis tools. To a subset of these experiments, paclitaxel was administered. This standard anti-cancer drug was used in 2 different concentrations to further increase the variability of the resulting data.

Four image stacks were obtained from each well. These stacks were subsequently transformed into maximum-projected images that retained the majority of topical information. The four images were then collated or “stitched” to form one single, large image that captures the entire well. This image data set was utilized mainly for testing and evaluating the various analytical methods. A total of 60 wells were imaged per plate, with two replicate wells for each patient-treatment-combination. This experimental design is illustrated in Supplemental Fig. 1. The quality of the images varied, and images from 4 wells were excluded for being largely out-of-focus (missing wells in the figure).

### Local entropy filtering accompanied with global thresholding provides optimal results in comparison to manual ground truth

**Figure 3:**
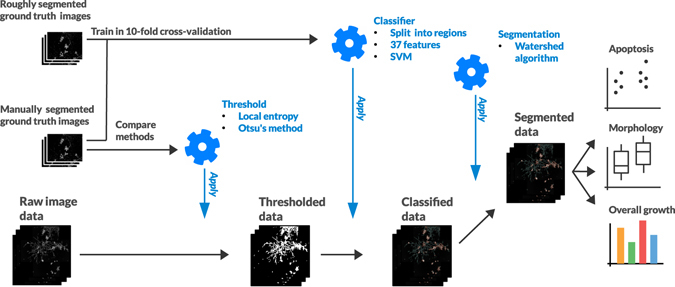
The complete chain of computational methods and their derivation. Below, the data processing steps are illustrated starting from the raw image data and ending with the classified and segmented data. Above, the development of the related methods is shown and their points of application are specified. The raw data are first thresholded using Otsu’s method on the local entropy filtered images. This approach was evaluated using a manually generated ground truth. The thresholded data are then classified between the two cell types with the SVM trained using 10-fold cross-validation. The data are then segmented into individual cell structures using the well-known Watershed algorithm. Finally, the overall growth, morphology and the number of very small structures are subjected to statistical analysis.

**Figure 4:**
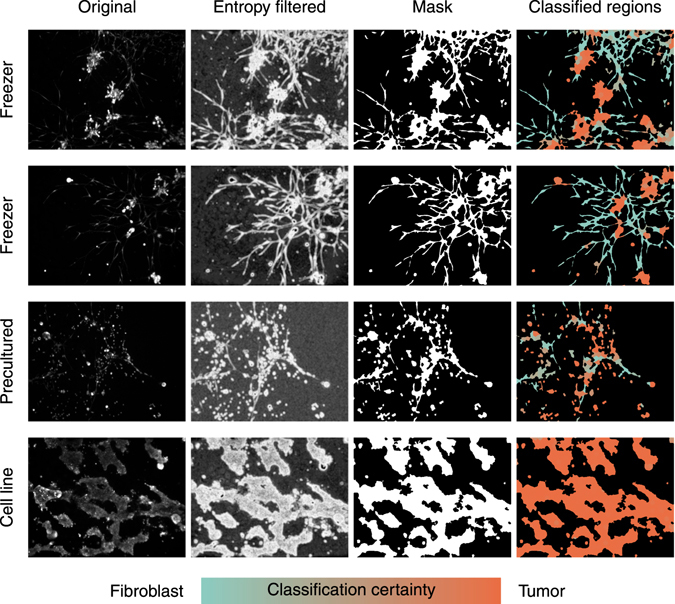
The suggested segmentation sequence and subsequent steps enable classification of different cell types in confocal images from PDX microtissue cultures. The original calcein-labeled live cell images, consisting of multicellular tumor organoids and fibroblasts were first local entropy filtered. Next, their foreground was separated from the background to obtain the segmentation masks. These masks were then split into individual regions that were subsequently classified into the two main cell types trained for. These were shown with distinct colors (Tumor cells: orange, CAFs/Fibroblast: blue). The color shading indicates the certainty of the classification. Cultures started directly from frozen cultures, from pre-cultured cells or by the use of cell lines, illustrate the impact of controlling the amount of fibroblasts included into 3D cultures. These example images were not part of the ground truth training set.

Next, the computational methods utilized in the study were evaluated. A full illustration of the image processing and the following statistical analyses is displayed in Fig. 3 while examples of the image data at various steps of the procedure are shown in Fig. 4. As a logical first step in the analysis, representative image areas that contained characteristic tumor tissue structures needed to be identified. This was achieved with thresholding, meaning the separation of the pixels that belong to the organoids or individual cells from those pixels that belong to the background (Fig. 3 left). Compared to standard 2D cultures of cell lines imaged by fluorescence microscopy, the complex multicellular structures found in the PDX microtissue data pose a considerably more difficult thresholding task. As observed in the example images in Fig. 4, some key characteristics needed to be considered when applying thresholding methods on these PDX cultures: 1) the images consist of a dark background with bright foreground, 2) the brightness of the objects varies greatly across and within the images. This depends 3) on the efficacy of live-cell staining as well as the position of objects within the stack of confocal images, and 4) the amount of content in each image varies greatly from practically empty images to images almost fully covered by structures. Given these extreme characteristics, conventional thresholding procedures are unlikely to reach optimal results. Thus, we carefully considered the choice of different thresholding methods, which may be suitable for our complex PDX cell models.

We propose a thresholding method based on local entropy filtering with an automatically obtained threshold value. This filter has been successfully used as a basis for the detailed 3D segmentation of 3D co-cultures of cell lines^25^. The same method can also be applied directly for thresholding of more complex patient-derived 3D cultures, permitting a higher throughput context. The local entropy filter places large values on pixel neighborhoods with high variability in pixel intensities. In contrast, regions with entropy close to 0 have only a few unique pixel values. In the context of fluorescence microscopy images, it is thus natural to assume that the image foreground should have higher entropy than the background. Examples of entropy-filtered images shown in Fig. 4 clearly support this assumption. Importantly, local entropy does not depend on either the local or global brightness of the images, but only on the variability among pixel intensities, thus addressing the heterogeneous brightness conditions. This standardizing effect is clearly visible in Fig. 4, where both bright and darker structures are equally illuminated.

Next, two widely used thresholding methods, one adaptive and one global, were tested: Otsu’s and Tsai’s methods. Otsu’s method^29^ is a popular clustering-based approach that separates the background and foreground pixels by minimizing the weighted sum of variances in the two classes. The method is parameter-free and adaptable to cases, in which the ratio of foreground versus background varies strongly^30^. Tsai’s method^31^ finds a threshold value that maintains the three first central moments of the pixel intensities as close as possible. This method does not have tuning parameters and is adaptable to distributions with varying shapes.

Both thresholding methods were applied to the original images and to the local entropy filtered images, in order to demonstrate the advantages or disadvantages of the filter. Each method was followed by a set of additional operations that take the data-specific conditions and existing prior knowledge into account. The full process then consisted of the following five steps: i) obtain the image mask by applying the chosen thresholding method, ii) place pixels with a grayscale value larger than 0.2 in the original image to the foreground, iii) apply a thinning algorithm to the mask, if the thresholding was applied to local entropy filtered image, iv) set any structures with maximum diameter of less than 10 pixels to the background, v) set any holes in the foreground smaller than 100 pixels to the foreground. In step ii, we took advantage of the prior knowledge that bright pixels always belong to the foreground. The boundary of 0.2 is very conservative but still benefitted the segmentation of very bright and smooth objects that could be missed by the entropy filter. Another drawback of the entropy filter was that it has the tendency to expand the structure boundaries. To correct for this trend, a thinning algorithm^32^, was applied to the obtained foreground in step iii). Importantly, this algorithm maintains the topological properties of the mask and no structures were lost or cut in two. The details of the algorithm are presented in Supplemental methods. In steps iv) and v), unrealistically small structures such as cell debris and holes in the organoids, were removed.

**Figure 5:**
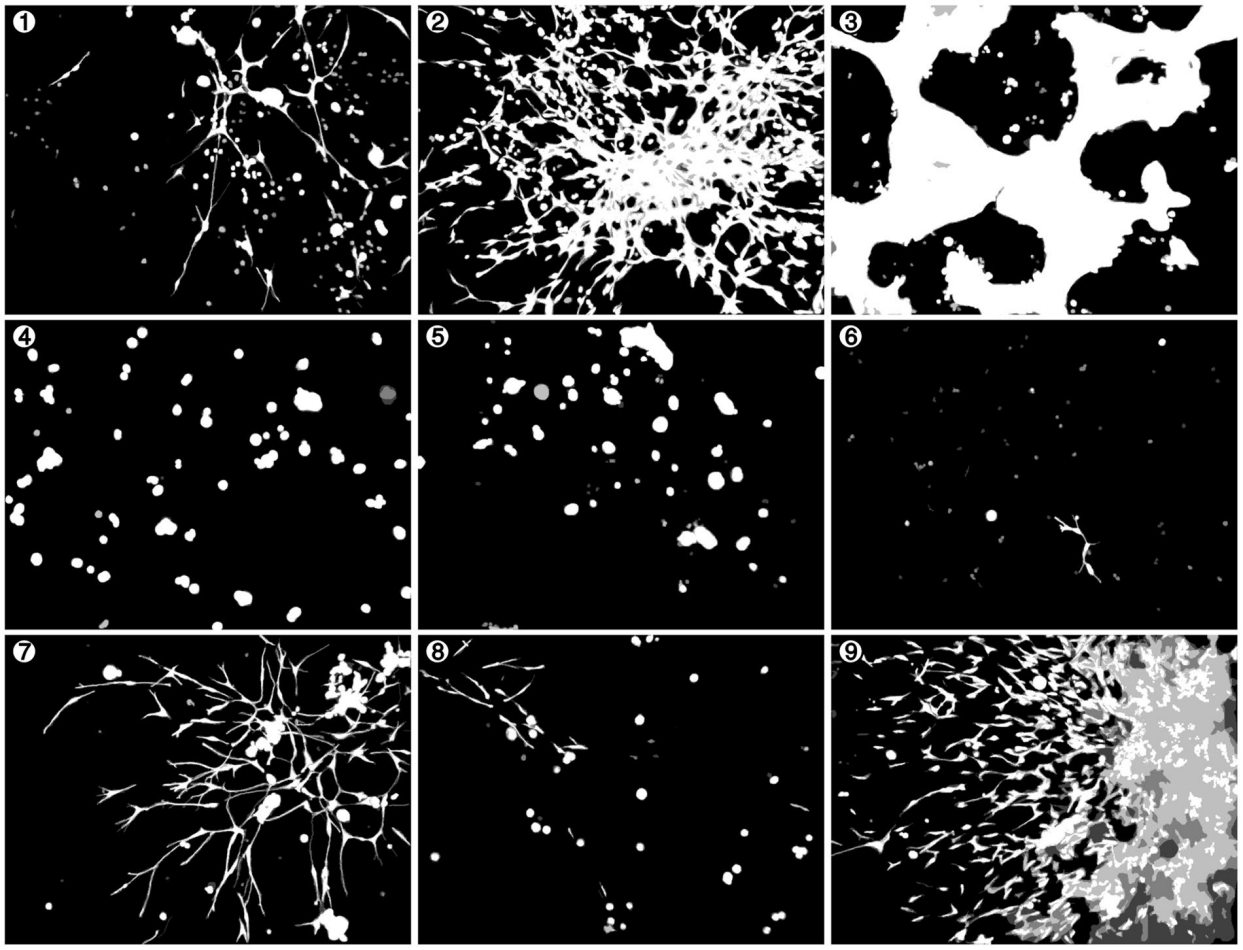
The manual thresholding for nine samples of confocal images averaged over the four experts PDX microtissue cultures are labelled in white (both tumor and stroma). White and black colors indicate a perfect agreement among the experts, whereas gray shades result from differing pixel assignments.

The different thresholding methods were tested with a representative sample of nine confocal images, one selected from each combination of patients and culture method. Each image was manually thresholded by four individual cell biology experts, according to the information obtained from the microtissue characterizations (Fig. 2). This allowed additional investigation of expert-to-expert variation in this task, on top of the generation of reference images. In addition, the observed disagreement between manual human segmentations can be used to set a realistic reference for the accuracy of the automated methods. To obtain a single ground truth for each image, the manual thresholding results were averaged over the experts, resulting in the nine images displayed in Fig. 5. The performance of the thresholding methods was then evaluated against these reference images. The original images are shown in Supplemental Fig. 3.

**Figure 6:**
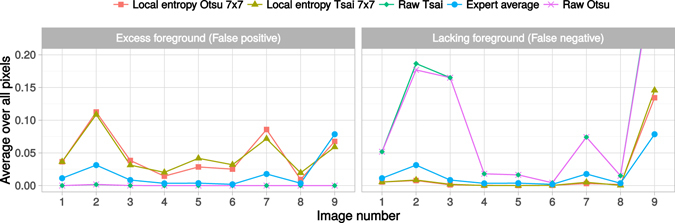
Comparison of the thresholding methods. The *Expert average* measures the variation between the experts and gives a reasonable bound for the obtainable results.

To compare the automatically obtained foregrounds to the reference images, continuous analogues for false positive (FPR) and false negative (FNR) rates were defined:

1 $$FPR=\frac{1}{N}\sum _{i}{({f}_{i}-{r}_{i})}_{+}\quad FNR=\frac{1}{N}\sum _{i}{({f}_{i}-{r}_{i})}_{-},$$

where *N* is the total number of pixels in the image, *i*∈{1, …, 672} × {1, …, 512} is the pixel index and *f*_*i*_ and *r*_*i*_ denote the pixel intensities in the automatically thresholded image and in corresponding reference image. FPR thus measures the average amount of excess foreground, compared to the reference image while FNR relates to the lack of foreground. The measures were calculated for each of the nine reference images, resulting in a total of 18 measures for each method candidate as displayed in Fig. 6.

All methods performed relatively well in terms of precision and did not falsely identify many background pixels as foreground (Fig. 6). The methods based on local entropy were more liberal in this regard, whereas the methods applied to the raw data remained very conservative. This conservatism was reflected in the FNR, where these two reference methods were in some cases having difficulties to capture the full foreground. The expert average FPR and FNR revealed that some images, namely 2, 7 and 9, were significantly more difficult to segment than the others and the expectations placed on the automated methods should thus be relaxed accordingly. Overall, the methods that included the local entropy filtering were preferable, despite the moderately increased FPR. Although the differences between the two candidates were not as pronounced, the method utilizing the Otsu’s threshold arguably provided the best overall results. The chosen method has an overall good performance with both the FPR and FNR below 10% in every image, except the very difficult image number 9.

### Local texture features allow accurate classification of the PDX microtissues

The two cell types, tumor cells and fibroblasts, typically form complex, interlaced superstructures (Fig. 4). Depending on their genotype and stimuli provided by their microenvironment, the tumor cells generally form organoids that can be either rounded or form grape like structures, in some cases even “stellate” structures with invasive “appendages”. In contrast, the fibroblasts are typically loosely grouped, rarely form cell clusters, and stretch out to form a more or less dense meshwork of cells throughout the entire cell culture area. Without any additional color labels to separate the cell classes from each other, and only gradual differences in live cell labeling via exposure to the Calcein AM dye, we cannot expect direct and simple segmentation approaches such as the method utilized by Di *et al*.^33^ to yield convincing results. Therefore, we first split the obtained image foreground into disjoint regions, which we then aimed to accurately classify into either the fibroblast/CAF or tumor cell type in a supervised manner (Fig. 3 middle). Conventional segmentation techniques, such as those used in^23, 34^, can then be applied to the labeled foreground, and allow a straightforward analysis of multicellular (tumor) or individual (fibroblast) cell structures.

In addition to the nine detailed ground truth images, a set of 18 only roughly segmented images curated by just one expert, was added to increase the size of the classifier training set to 27 images. These additional training images separate the foreground into the two cell types, but do not accurately portray the boundaries of multicellular structures. Generating such training material was relatively straightforward and fast, thus allowing the classifier to be retrained for new data with manageable effort.

In the PDX microtissues, single, most likely dead and/or apoptotic cells demonstrated a small and round phenotype regardless of their cell type. Differentiating between fibroblast and tumor cells in these cases was not attempted; and it is likely that both cell types are presented in this group. Instead, small objects with a maximum diameter of less than 30 pixels and roundness of at least 0.5 (50% roundness compared to ideal sphere) were excluded from the training and simply labelled as “rounded single cells”. However, these data were not excluded from the subsequent analyses, but were displayed as a third class of cells. The roundness measure is defined in detail later in the section describing the statistical analyses.

After the removal of single “dead” cells, the foreground of the 27 training images was identified using the proposed thresholding method and then split into individual disjoint Voronoi regions, using a propagation algorithm described by Jones *et al*.^35^. Importantly, the regions obtained with this algorithm respect the borders of the foreground but their general shape and size can be controlled via the supplied seed pixels of the regions. For optimal segmentation results, the regions should be large enough to retain the amount of information necessary for the classification, but still small enough to allow accurate segmentation. Here, we opted to use hexagonal regions of approximately 350 pixels, roughly the size of one large cell or a small multicellular structure. An example of the resulting (and classified) regions is shown in Fig. 4. A total of 2851 regions labeled as “fibroblast” and 2739 as “tumor” were obtained from the 27 images.

The ground truth labels for the regions were decided with a majority vote over the manually labeled images: the total number of pixels colored as cancer and as fibroblast in the region across all ground truth images was calculated, after which the label of the region was set to which ever total was higher. For the large majority of the regions (85%), the decision was close to unambiguous with one of the two labels getting over 90% of the votes.

A set of 37 numerical features describing the texture of each region was extracted:

- Standardized entropy of all the pixels in the region calculated in the same manner as for the individual windows in the thresholding phase (1 feature)
- Standard deviation of the pixel values in the region (1 feature)
- Local binary pattern histogram (LBP)^36^ from 3 × 3 windows in the smallest form mapping with non-uniform patterns grouped under one label (9 features). LBPs are a powerful family of texture features that utilize a small set of codes that relate to local pixel neighborhood characteristics such as edges, corners and rough surfaces. The relative frequencies of these codes is calculated for a given region, forming the final feature vector. Here, we extracted a total of 10 binary patterns and thus a 10-dimensional feature vector.
- Haralick features^37^ (26 features). These features are a well-established set of image texture descriptors based on the symmetric pixel co-occurrence matrix *C*. The entries *C*(*i*,*j*), *i*,*j* = 0, …, 255 denote the probability that two randomly picked adjacent pixels in the image have the values *i* and *j*. The actual features are various properties of the co-occurrence matrix such as the energy, entropy, or correlation. A total of 26 such features are readily available and documented in the EBImage^38^ R-package^39^.

Importantly, these features do not depend on the alignment, or the overall brightness of the regions. Some of the features mentioned above were very highly correlated, to the extent that they caused problems in the subsequent classifier training phase. This redundancy was tackled by substituting the features by a set of their principal components^40^. The features were first standardized to have a mean 0 and variance 1 for equal weighting, after which the first 23 principal components were extracted. Together, these components accounted for 95% of the variance in the data. Finally, the culture method was added as a categorical variable among the principal components.

A set of classifiers was considered:

- logistic regression with main effects^41^
- random forest (RF)^42, 43^ with 1000 trees and $\sqrt{24}\approx 5$ components sampled at each split. The size of the trees was controlled by varying the minimum proportion of observations allowed in the terminal nodes of the trees, specific values being 2.5%, 5% and 10%.
- support vector machine (SVM)^44^ with a Gaussian kernel. The hyper parameter *σ* controlling the Gaussian kernel’s shape was estimated automatically from the data using the method implemented in the R package *kernlab* ^45^. The SVM algorithm is controlled by a cost parameter *C* for which we considered values 1,10 and 100.
- A bundle of logistic regression, using 5-nearest neighbors and 10-nearest neighbors. Bundling combines the ideas of ensemble learning^46^ and bootstrap aggregation, or bagging^47^, of decision trees and has often been found to produce superior results when compared to single learners. A bootstrap sample is taken from the training data and the out-of-bag samples are used for training the chosen ensemble of learners. The predictions of these learners are supplied as additional covariates to a decision tree that is then trained on the in-bag samples. A total of 20 such bootstrap iterations are run, after which the final prediction is obtained as an average over the 20 trees.

The performance of the classifiers was assessed with a 10-fold cross-validation^48^. The results for each cell culture variation are shown in Table 1. Only moderate differences were observed between the methods, however the overall results seem to favor the SVM classifier, which was chosen as the final model. The overall accuracy of approximately 80% can be considered as solid performance in such a complex image classification task and good enough for obtaining sufficient accuracy for subsequent analyses. The ROC-curves of the SVM model and additional, more detailed characteristics are illustrated in Supplemental Fig. 4. The trade-off between a classifiers sensitivity and its specificity can be controlled by adjusting the decision threshold of the predictions. Since there was no reason to favor one cell type over the other, a threshold that results in equal sensitivity and specificity was used. Finally, the chosen classifier was used for estimating the total growth of the microtissues displayed in the Supplemental Fig. 1.

**Table 1:** Cross-validation results for the different classifier candidates.

method	Freezer N = 382/143				Precultured N = 1946/294			
	AUC	ACC	F1	SensSpec	AUC	ACC	F1	SensSpec
Logistic regression	85.1	77.5	65.3	77.6	89.1	81.7	53.9	81.6
Random forest	87.8	80.4	69.1	80.4	86.3	78.8	49.5	78.9
SVM	92.0	82.7	72.2	82.5	88.2	79.9	51.1	79.9
Bundle	88.4	78.3	65.9	76.9	88.4	80.8	52.6	81.0

The results are displayed separately for the two culturing methods that are relevant to the task. The metrics are *AUC* = area under the ROC curve, *ACC* = Accuracy, *F1* = the harmonic mean of precision and recall, and *SensSpec* = the sensitivity and specificity of the classifier. Images of cells cultured as cell lines do not contain any fibroblasts and are classified perfectly by each of the tested classifiers. The best variation of each method was chosen based on the area under the AUC. Reference class for the metrics is cancer.

### The computational methods generalize to cell line co-culture data

To validate the proposed image analysis method and to test its applicability to cell line cultures, we refer to an image data set previously published by Åkerfelt *et al*.^16^. The data set consisted of organotypic 3D co-cultures, using fluorescence-tagged cell lines. Specifically, the co-cultures comprised of fluorescent prostate cancer cell line LNCaP (stably expressing the dsRed marker) and the non-transformed PF179T fibroblast cell line, expressing green-fluorescent protein (GFP). Importantly, the stably expressed fluorescent labels separated the two cell types clearly into different color channels. The image data set included a total of 9 drug treatments in two concentrations, and a DMSO vehicle control. The same ECM composition of Matrigel and type I collagen, as well as the same type of live cell confocal imaging was used as in our PDX experiments.

The original red-and-green images were transformed to grayscale using the luminance preserving transformation: 0.2126*R* + 0.7152*G* + 0.0722*B* in order to mimic the black-and-white PDX data. 9 accurately segmented ground truth images were generated to measure the accuracy of the proposed thresholding method, while the SVM classifier was trained on the full set of color-separated images.

Our proposed thresholding method–in the absence of labeled marker expression–achieved an overall accuracy of 94.1% with FPR and FNR equal to 5.0% and 0.8% correspondingly. The overall cross-validated accuracy of the trained classifier was measured to be 92.2% with an AUC of 97.3%. Both results were highly satisfactory, demonstrating that the method can also be successfully applied to microtissue culture data of traditional cell lines. Examples of segmented images from these data are shown in the Supplemental Fig. 5.

### High-content image analysis of anti-cancer drug treatments on PDX microtissues

Next, a small-scale drug chemosensitivity test was performed using standard-of care drug treatments for NSCLC. PDX cell suspensions from two selected patient-derived xenografts (LXFA 923 and LXFA 1647), which were selected due to their robust microtissue formation in 3D cultures, were cultured using the “pre-culture” method; meaning excess fibroblasts were removed prior to seeding into ECM. These PDX microtissues were then treated with cisplatin, docetaxel and gemcitabine, in three different concentrations. Here, our focus was shifted towards the estimation of drug responses and interpretation of the phenotypic responses of the drug treatments. One 60-well plate was prepared for each patient, resulting in a total of 120 imaged wells. The design of this study is shown in Supplemental Fig. 2. After 12 days of organotypic 3D culture, the PDX microtissues were imaged using live cell confocal microscopy.

To first test the generalizability of the classifier, rough, manually segmented images were generated from this new set of confocal images. One well was picked from each patient-drug-concentration combination, resulting in 20 curated ground truth images. Regardless of the nominally equal experiment conditions, the trained classifier did not generalize well concerning the second PDX data set. The observed initial accuracy with the chosen decision threshold value was only an unsatisfactory 74.4%. This indicated that the classifier was still sensitive towards small uncontrollable variation between the experiments and its performance should be checked before applying to new data. The classifier was retrained using the newly generated ground truth images, after which it reached 84.3% overall accuracy and 89.8% AUC.

After the PDX microtissue classification, the detected tumor and fibroblast foregrounds were further segmented into individual structures. This was achieved using a previously described method^23, 34^, by applying the watershed algorithm^49^ to the distance-transformed mask of the foreground (Fig. 3 right). In practice, this approach separates structures that are connected only by a small number of pixels.

### Statistical analysis of PDX microtissue growth and structure morphology shows differences between individual patient samples

**Figure 7:**
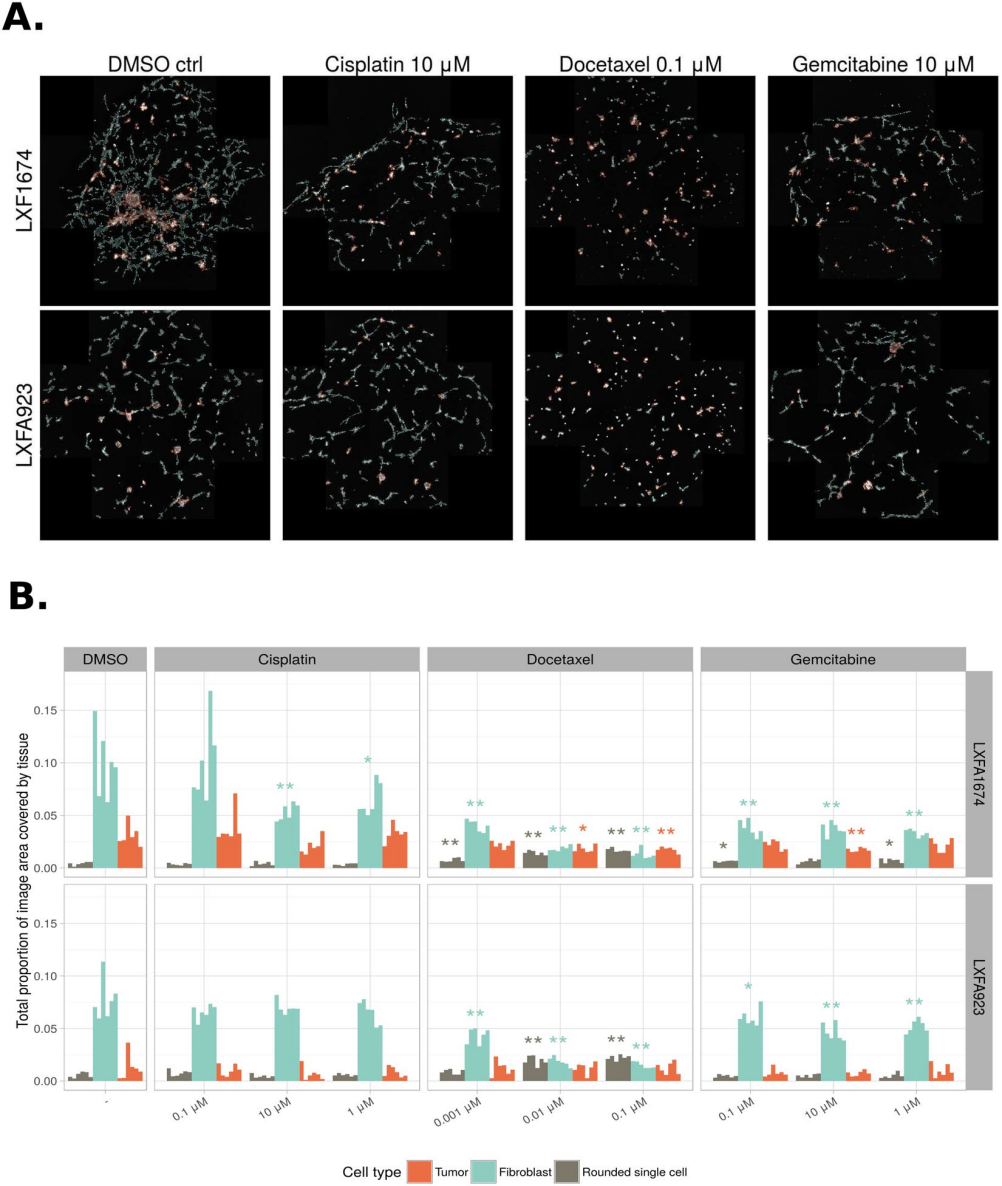
Segmentation and quantification of drug responses in PDX microtissues. (**A**) Examples of original segmented images from two patient samples (LXFA 1647 and LXFA 923) treated with standard-of-care drugs, cisplatin, docetaxel and gemcitabine. Only example segmented images of the highest drug concentrations are shown. Different cell types are depicted by distinct colors. (**B**) The estimated area covered by the three cell types: tumor, fibroblast and rounded single cells. The data are based on the estimates given by the proposed segmentation sequence. Each bar corresponds to one well. The stars denote the p-value of the robust Van den Waerden test^63^ against the DMSO control: *p < 0.05, **p < 0.01, ***p < 0.001.

By thresholding and classifying the foreground of the PDX data, we obtained an overview of the growth of the PDX microtissue cultures, and how this was affected by the different drug treatments (Fig. 7A). The estimated total growth of tumor cells and fibroblasts, as well as the number of rounded single dead or dying cells, is shown in Fig. 7B. Surprisingly, cisplatin did not show a significant effect on any cell population, and only slightly reduced the fibroblast growth in patient sample LXFA1647. Docetaxel was clearly the most effective treatment, and it significantly reduced the growth of tumor organoids (4–28%) and fibroblasts (44–59%), already at low concentrations (1 nM), compared to the control treatment. In addition, a marked increase of rounded single cells was detected in both patient samples, implying a significant increase in cytotoxicity and cell death. Gemcitabine was able to block both tumor organoid and fibroblast growth in patient sample LXFA 1647 at a higher concentration (10 μM), while only fibroblasts were affected in patient sample LXFA923 (Fig. 7B). These results suggest an individual drug response pattern in the different patient samples. For this reason, we decided to conduct a detailed investigation of the individual tumor organoid morphology, in order to better understand the effects of the different treatments.

**Figure 8:**
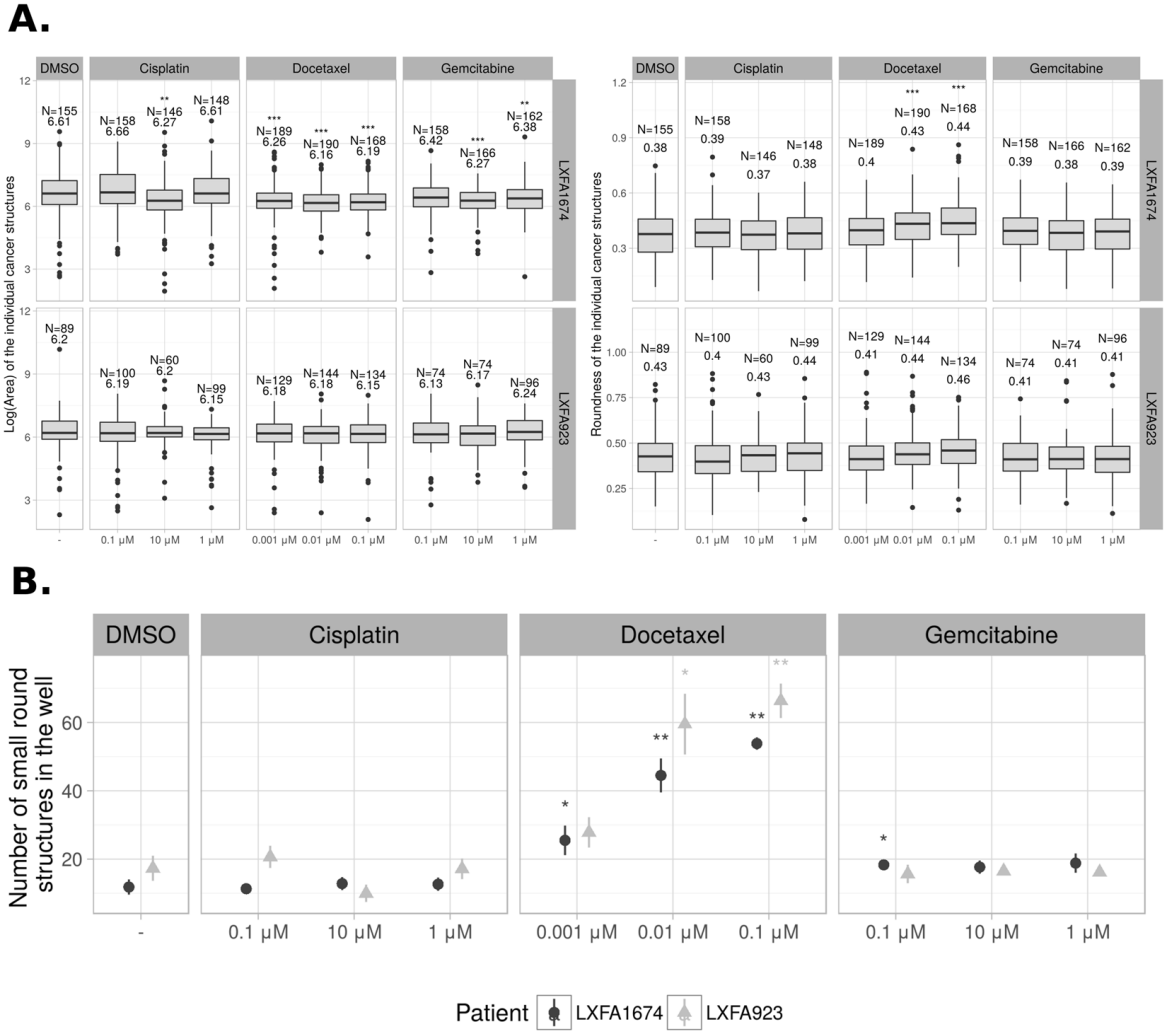
Morphometric analysis of tumor organoids. (**A**) The distributions of area and roundness of the tumor organoids, treated with standard-of-care drugs. Two patient-derived xenograft samples, LXFA 1647 and LXFA 923, were treated with standard-of-care drugs, cisplatin, docetaxel and gemcitabine in three different concentrations. Only tumor organoids were included and single rounded, dying cells were excluded from the analysis. The stars denote the p-value of the robust Van den Waerden test^63^ against the DMSO control: *p < 0.05, **p < 0.01, ***p < 0.001. The sample size and the median value are displayed on top of the graphs. (**B**) Quantification of single, rounded cells that indicate cell death, across drug treatments. Mean and its standard error are shown. The stars denote the p-value of the robust Van den Waerden test^63^ against the DMSO control. Sample size in each group is 6.

The distributions of two morphological features of the tumor organoids, *area* and *roundness*, across the patient samples and treatments were measured and are illustrated in Fig. 8A. Area measures the growth and thus proliferation of the living tumors, while roundness indicates the level of epithelial differentiation, which typically leads to pronounced cell-cell-contacts between tumor cells and therefore rounded organoids. The Roundness measure indicates the opposite of tumor invasion, as well-differentiated organoids are typically surrounded by an intact basement membrane, which effectively blocks invasive tendencies. Increased roundness can also be the result of growth inhibition and is therefore often observed in small or effectively drug-treated organoids. The tumor area is simply measured as the number of pixels in the structure, while roundness is obtained as a function of the area (*A*) and the maximum structure diameter *D*: 4*πA*/(*πD*)^2^. Roundness is thus a measure of a structure’s deviation from a perfect circle, where larger values indicate greater circularity. Here, we explored the effects of different treatments in the two patient samples (Fig. 8A). Docetaxel significantly reduced the size and increased the roundness of the LXFA 1674 tumor organoids, resulting in smaller and rounder shapes compared to the DMSO control. This demonstrates that both tumor organoid growth and invasion were effectively blocked with docetaxel, as irregular structures became smaller, they also became more round. In contrast, the effects of both cisplatin and gemcitabine were not pronounced: significant growth reduction was only detected at the highest concentration of both drugs in patient sample LFXA 1647. Cisplatin and gemcitabine did not block invasion, indicated by no increase in the roundness of the organoids in any of the patient-derived xenograft samples.

To measure the possible increase in cell death caused by the treatments, we also calculated the frequency of the non-classified single, small and rounded structures in each well. These small rounded structures are indicative of dead and dying cells. The average numbers of round cells are shown in Fig. 8B. A dramatic effect was observed for both patient tissues with docetaxel treatment, demonstrating a clear increase in the response at higher drug doses. These results imply that although docetaxel treatment effectively blocks tumor growth and invasion, it also has a high level of cytotoxicity in both patient samples at high concentrations (Fig. 8A). In contrast, cisplatin and gemcitabine showed almost no cytotoxic effects (Fig. 8B) even at higher concentrations, but they also failed at blocking tumor cell invasion (Fig. 8A).

### A correspondence in drug response between *in vitro* and *in vivo*

**Figure 9:**
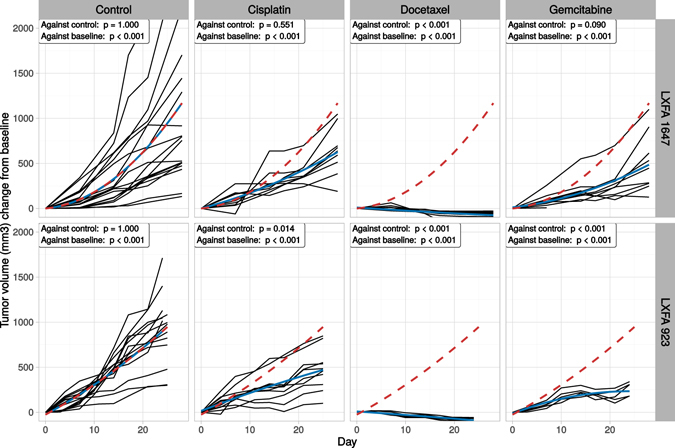
Drug sensitivity tests in PDX models. Two PDX models, LXFA 1647 and LXFA 923, were treated with standard-of-care drugs, cisplatin (5 *mg*/*kg*/*day* for LXFA923 and 8 *mg*/*kg*/*day* for LXFA 1647), docetaxel (150 *mg*/*kg*/*day*) and gemcitabine (200 *mg*/*kg*/*day*). Each black line represents repeated measurements of tumor volume from an individual mouse, blue solid line the treatment average and red dashed line the control average. The average fits are obtained with a simple second degree polynomial. Two statistical tests are performed: one against the control treatment and one against the baseline. The statistical tests were performed using robust and non-parametric methods for repeated measurements^62^. These methods are less vulnerable to the heterogeneous variance and variable trends of the growth curves compared to conventionally used parametric models.

To further validate the results obtained in the *in vitro* PDX microtissue models, the drug responses were compared to the responses in *in vivo* PDX tumors utilizing the same set of drug treatments. Tumor material from patients LXFA 1647 and LXFA 923 was implanted into mice and observed for 25 days. The efficacy of the administered drugs was measured as the reduction in tumor volume over time. The results of the *in vivo* PDX experiments were in agreement with our previous findings in the PDX microtissue models, thereby validating the *in vitro* results (Fig. 9). Only Docetaxel (150 *mg*/*kg*/*day*) demonstrated a strong tumor growth inhibitory effect, while the efficacies of cisplatin (5 *mg*/*kg*/*day* for LXFA923 and 8 *mg*/*kg*/*day* for LXFA 1647) and gemcitabine (200 *mg*/*kg*/*day*) were found to be less consistent. An *in vivo* measurement for the toxicity of the drug treatments was obtained by observing the weight of the mice throughout the study (Supplemental Fig. 6). In addition, docetaxel was the least toxic drug at the organism level, as it was the only treatment in addition to the control that displayed a positive weight growth. Although *in vivo* toxicology cannot be directly compared to *in vitro*, docetaxel was found to be the most toxic substance at the cellular level (Fig. 8B). Taken together, our results indicate that a combination of both *in vitro* and *in vivo* PDX models may be a suitable strategy for predicting the drug sensitivity in cancer patients, as the 3D cultures predicted the outcome of mouse drug exposures.

## Materials and Methods

### PDX cell suspensions

The PDX NSCLC cell suspensions were obtained from Charles River Discovery Research Services Germany GmbH. Primary tumor cell pieces were originally isolated from the three different patients, and were then amplified for 9–20 passages as mouse xenografts. Patient donor tissue was obtained during surgery and implanted within 2–3 hours subcutaneously under isoflurane anesthesia in NMRI nude mice (Charles River, Germany). When tumors develop, mice were sacrificed and tumor tissue fragments re-implanted into recipient mice of the same strain.

### Cell suspensions and cell lines

All three NSCLC PDX cell suspensions and PDX cell lines were cultured in DMEM medium (Sigma-Aldrich) supplemented with 10% FBS, 1% penicillin/streptomycin and 1% L-glutamine, 25 mM Hepes, and 1% non-essential amino acids. Prostate cancer cell line LNCaP and PF179T cancer associated fibroblasts (CAFs) were both propagated in RPMI-1640 medium (Sigma-Aldrich), supplemented with 10% FBS, 1% penicillin/streptomycin and 1% L-glutamine.

### Miniaturized organotypic cultures in ECM

All organotypic cultures were performed in 96-well angiogenesis plates (Ibidi)^23^. These plates allow cells to be embedded in a defined and narrow focal plane between two layers of ECM. The volume of a single 3D experiment requires only 30 *μl* of ECM: growth factor-reduced Matrigel (Corning), stock 8 mg/ml and type I collagen (Corning), stock 3 mg/ml, or a 1:1 mixture of both. The pH of collagen solution was neutralized with NaOH prior to use. Organotypic co-culture that generates microtissues were prepared as described previously^16^. In short, bottom wells of the plates were filled with 10 *μl* of 50% ECM in medium. Single cell suspensions were mixed with 25% ECM in medium and 20 *μl* was placed on top of the polymerized bottom gel at a density of 1000 cells/well. Hence, one tumor organoid is typically generated from a single cell (clonal approach). The wells were then filled with medium, which was changed every 2nd – 3rd day.

### Live-cell monitoring using Incucyte FLR

Live-cell imaging was carried out in a systematic, standardized fashion. Incucyte FLR live-cell fluorescent imaging device (Essen Bioscience) was utilized for continuous monitoring of 3D culture for up to two weeks. These images were based on phase contrast microscopy.

### Live cell staining and confocal image acquisition

The organotypic cultures were stained with live cell dye Calcein AM to visualize living cells (Thermo Fisher Scientific). At the endpoint, day 12 of organotypic culture, 3D confocal images were acquired with a Zeiss Axiovert-200M microscope, equipped with Yokogawa CSU22 spinning disc confocal unit using Zeiss Plan-Neofluar 5x objective. Maximum intensity projections and subsequent background noise removal were performed with SlideBook 6 (3i Intelligent Imaging Innovations Inc.).

### Confocal image data pre-processing

In each data set, four image stacks of 16 layers were obtained from each well using confocal imaging. The stacks were then max-projected into 240 8-bit grayscale images with a resolution of 672*x*512 pixels, each pixel having a value between 0 and 1. However, instead of imaging four completely separate regions, these images have a slight overlap, the amount of which varies from well to well. On average, 27% of the total number of pixels within the images were redundant in each set. To avoid analyzing duplicated data, the four images were collated together to form a single well-wide image. This is achieved by finding the phase correlation of their fast Fourier transforms as described for instance by Reddy and Chatterji^50^. For the sake of clarity, the figures of this study contain mostly original 672*x*512 pixel images, however all statistical analyses were performed on the collated images.

### The local entropy filter

The local entropy filtered value *e*_*i*_ for a pixel *x*_*i*_, *i*∈{1, …, 672} × {1, …, 512} is defined as the observed entropy in a neighborhood *w*_*i*_ around the pixel:

2 $${e}_{i}=\sum _{v\in V}{p}_{v}{\mathrm{log}}_{2}({p}_{v}),$$

where *V* is the set of all unique pixel values in *w*_*i*_ and *p*_*v*_ is the proportion of pixels with value *v* in *w*_*i*_. For windows that exceed the image borders, the amount of pixels available for calculating the entropy is reduced. To obtain a measure that does not depend on the number of pixels, we used a standardized version of the local entropy:

3 $${e}_{i}^{\ast }={e}_{i}/{\mathrm{log}}_{2}(\#{w}_{i}),$$

where #*w*_*i*_ is the number of pixels in the neighborhood *w*_*i*_ and log_2_(#*w*_*i*_) is the maximum obtainable entropy for #*w*_*i*_ pixels. The score was thus standardized on the interval [0, 1]. A window size of 7 × 7 pixels was used, reflecting the low resolution of the images.

### Immunofluorescence of organotypic cell cultures

The organotypic cultures were fixed in the *μ*-angiogenesis wells with 2% paraformaldehyde (PFA) in PBS for 20 min, washed with cold PBS and blocked in 20% horse serum. Cell permeabilization was performed by addition of 0.7% Triton X-100 (Sigma-Aldrich). Fixed cultures were incubated at 4 °C overnight with primary antibodies (1:100), washed with PBS and incubated for 3–4 h at RT with secondary antibodies (1:500) and Draq5 (1:1000, Cell Signaling Technology), to visualize the nuclei. The following primary antibodies were used: anti-E-cadherin (HECD-1, Abcam), and anti-smooth muscle actin (sc-53015, Santa Cruz). Images were taken with the Zeiss Axiovert-200 M spinning disc confocal microscope, using a 20x objective. Z-stacks were acquired with a step-size of 8 *μ*m. The images were pre-processed using SlideBook 6 and NIH Image J.

### Drug treatments of *in vitro* organotypic cultures

All drugs were dissolved in DMSO. Standard-of-care drugs were selected for treating the organotypic PDX cell cultures; Paclitaxel, Cisplatin (Selleck), Docetaxel and Gemcitabine (Tocris). Two or three different concentrations of each drug were utilized, and all drug exposures were performed in triplicate wells, including vehicle control. Drug treatments were initiated after 4 days of 3D culture, when significant organoids had already formed, and continued for an additional 8 days.

### Drug treatments of *in vivo* PDXes

For treatment experiments, NMRI nude mice (Charles River, Germany) were implanted subcutaneously with fragments of LXFA 923 or LXFA 1647 tumors. When median tumor volume reached 100–200 *mm* ^3^, individual animals were stratified into different treatment groups (group size n = 5). Mice received either: Control Vehicle (NaCl, 5–10 ml/kg), Cisplatin (5 or 8 mg/kg/d, days: 1, 14; subcutaneously), Docetaxel (15 mg/kg/d, days: 0, 7, 14; intravenously) or Gemcitabine (200 mg/kg/, days: 0, 7, 14; intravenously). Tumor volume was determined by caliper measurement twice a week together with body weight determination.

### Ethical statement

Charles River Discovery Research Services Germany GmbH runs an AAALAC accredited animal facility. All experiments were performed in accordance with German law (license nr G13/13 Regierungspräsidium Freiburg) and the respective AAALAC guidelines. All patients gave their informed consent and their data were anonymized at the respective clinical institution. The study was approved by the ethical committee Freiburg, Germany.

### Data Availability

The datasets generated during and/or analyzed during the current study are available from the corresponding author on reasonable request.

## Discussion

In this study, we have explored the feasibility and biological relevance of organotypic cultures of primary cells isolated from PDX tumors cultures, and their reliability in predicting patient response to anti-cancer treatments. For this purpose, we developed a chain of image analysis procedures that initiate with the processing of the original, confocal microscope images, and is completed with the statistical analysis of morphometric or phenotypic data. We then show that these numerical measures obtained correspond to specific chemosensitivity and drug treatment effects.

Each step of the image analyses represents also a possible source of bias and error. In a hypothetical case involving a wealth of ground truth data and computation time, the optimal solution to the problem would likely be a single deep learning neural network^51^ that would simultaneously tackle both the important thresholding and cell classification tasks. However, given the practical limitations, such as limited computation time, a more tailored solution had to be designed. This was achieved by exploiting available problem-specific context knowledge where possible, and by splitting the task into multiple smaller and more tractable sub-problems. The performance of different thresholding methods was tested against manually generated ground truth data. While such validation is considered standard practice in image analysis, the comparison of multiple experts is a novel approach in the field of cell imaging and thus a valuable contribution on its own. Indeed, significant variation between the experts was observed in some images, clearly demonstrating the need for the multiple-expert-approach in future studies on similar data.

After thresholding, the obtained image foreground was split into local regions that were then classified between the two cell types based on (principal components of) a set of texture features. A more ambitious approach would consider classifying each pixel at a time based on its local neighborhood however this would significantly increase the required computation time. Furthermore, the local regions remain small enough to estimate of the boundary between the cell types with reasonable accuracy. Given the novelty of the PDX data, examples of such texture classification are not common in the literature. Approaches to supervised phenotypical classification of individual cells and cell structures do exist^19, 52, 53^, however our problem comes closer to separating between tissue types in *in vivo* images from histologic tissue sections^54, 55^. In contrast to PDX data, these sections are characterized by high resolution and abundant available training data. Specifically, the increased resolution allows for the utilization of rectangular local regions without a significant loss of accuracy and also simplifies the feature extraction. These differences made the direct application of the existing methods to our PDX data impractical.

The computational time required for performing the image processing is important, since this could be a limiting factor for performing larger scale phenotypic screening projects. The proposed methods are fast enough to allow high-throughput analyses, as the average time taken to threshold and classify one collated PDX well image was 31.8 seconds leading to an approximately 95 minute computation time for 180 images in the two data sets. This time can be significantly reduced if multiple CPUs are utilized. The calculations were performed using a single core on an Intel®Xeon®X5450 3.00 GHz processor.

After the initial image processing, both *in vitro* and *in vivo* treatment effect data were subjected to statistical analysis. Modern robust statistical methods were used throughout the analysis in order to mitigate the effects of possible anomalies (outliers) and to relax the usually applied assumption of normally distributed data. This ensures that the proposed methods can be applied as such for future datasets that might not have similar distributional properties. The mindset also applies more generally to the whole quantitative part of this study. The gathered methodology is efficiently applied both in terms of computational burden and manual labor. The chosen methods are also likely to generalize well to new similar data, although retraining the cell type classifier is recommended.

In addition to challenges related to accurate, quantitative analysis of *in vitro* cancer models that are currently utilized in drug discovery and pre-clinical chemosensitivity testing, another drawback is their poor or unknown biological relevance. In NSCLC, this is even more pronounced than in other cancer types^7^. In contrast to the majority of established cell lines, the use of patient-derived, primary cell cultures allow for personalized drug screens with increased clinical relevance. The similarity of PDX tumors with primary tumors has been reported repeatedly. PDX models show individual responses to both classical chemotherapy and targeted anti-epidermal growth factor receptor agents, just as NSCLC tumors in patients. PDX tumors maintain much of the original tumor heterogeneity and allow important studies that rely on the preservation of specific tumor cell subpopulations. This further indicates that in PDXs, modulation of such cell populations after drug treatment may be comparable to patient tumors^4^. In addition, the order of developing the *in vitro* PDX models is likely to be important. Primary tumor cell suspensions can, for example, be directly used for drug sensitivity testing in organotypic cultures^56^. The resulting organoid cultures can later be transferred into PDXes^57^. For some tumor types this approach has been successful^58^. However, the amount of primary tumor material available for implanting is often limited, original fibroblasts may get be lost, and for many tumor types it is still inefficient and technically challenging to establish 3D organoid cultures *in vitro*. This, in turn, limits the material that can later be used for successful generation of PDXes. Therefore, our approach may provide a straightforward and effective way to expand the patient-derived tumor population in the PDXes, prior to conducting drug sensitivity tests in the PDX-microtissues. This approach may also increase the likelihood that patient-derived materials from a larger fraction of patients will be available for precision medicine. During the amplification step, the tumors are also re-populated by mouse fibroblasts, which remain present as a stromal component in the PDX-microtissue *in vitro* models.

Overall, the response rates observed in PDX were well in accordance with the results of matching clinical studies^5^. Furthermore, PDX models show essentially the same genetic profiles of mutations, gains and losses of DNA^6^, although some minor genetic drift has been observed that may be due to *in vivo* selection after grafting. At least some mutations can show higher allele frequencies in the PDXs than in the original patient tumors. This suggests some possibility of tumor cell enrichment, changes in subpopulations and thus of altered heterogeneity in PDXs compared to primary tumors^59^, however, on a scale much lower than the vast numbers of mutations observed in cell lines that may accumulate after decades of culture *in vitro*. One of the key observations related to the biological relevance of PDX for human tumor biology is the rapid replacement of human fibroblasts by mouse stroma. PDX tumors maintain the human microenvironment only for a measure of weeks after engraftment. This is followed by complete substitution with murine stroma which occurs after only 2–3 passages in the mouse^4^. Nevertheless, there is plenty of indication that mouse fibroblasts replace the function of their human counterparts almost completely. Therefore, PDX remain a promising model even for studies of tumor-microenvironment interactions.

The major drawback for personalized chemosensitivity testing *in vitro* is the lack of standardized protocols. Primary tumor cell culture is hampered by many technical difficulties, including poor cell viability, weak growth, and contamination by host cells, especially fibroblasts. *In vitro* propagation may also change the ratio of original cell populations present in the tumor. Currently, no routine protocols exist that would circumvent all of these compromising technical difficulties. In breast cancers, successful initiation of primary cultures as well as PDX correlates closely with tumor aggressiveness and progression^60^, indicating a tendency towards enrichment of the most aggressive and proliferative cell populations in both methods. To establish primary tumor cell cultures of NSCLC, very few successful protocols have been published to date. Some of these involve a technique to consistently retain cell – cell contacts during all steps of preparation. After tumor biopsy specimens have been partially digested and cultured in a serum-free medium, the tumor fragments spontaneously form organoids^61^ and can be successfully maintained *in vitro*. The seeding of cell suspensions isolated from PDX tumors in our hands uses a similar principle, in particular the “freezer” strategy. Our results indicate that the isolation of tumor cell populations from PDX is technically less demanding and the amount of cells that can be used for screening is significantly increased compared to methods that rely entirely on fresh patient biopsies. Characterization of PDX tumors has convincingly indicated that the majority of genetic properties and cellular heterogeneity is retained or only slightly compromised.

PDX-derived cell suspensions were used for generation of *in vitro* organotypic 3D cultures, which were then used for drug chemosensitivity tests. This strategy required development of robust methods not only for standardizing cell- and tissue-culture protocols, but also for successful high-content imaging and subsequent image analyses. Accordingly, the experimental approach had to avoid the formation of dense, opaque and solid structures that cannot be effectively imaged by automated confocal microscopy. Therefore, all drug chemosensitivity assays were performed prior to the formation of dense tumor and stromal tissue structures. Our results from *in vitro* drug tests using PDX cultures demonstrated that docetaxel was the most effective drug, as it significantly reduced the overall growth of tumor organoids. Docetaxel targeted the fibroblasts already at low concentrations, but also the growth and invasion of individual tumor islands were effectively blocked. In comparison, both cisplatin and gemcitabine showed significant growth reduction only at the highest drug concentrations, and did not specifically block tumor cell invasion in any of the patient samples. Docetaxel was therefore the most effective treatment *in vitro*, as corresponding drug effects could be validated in the original PDX tumors *in vivo*.

We suggest that PDX-derived microtissues in 3D organotypic cultures may serve as a “missing link” between *in vivo* PDX models and classic 2D cell-culture based drug sensitivity assays. This approach opens new opportunities for early stage drug discovery and lead optimization. PDX microtissues may potentially bridge the gap between relatively high experimental throughput and low costs of phenotypic screening methods, compared to the notoriously cost-intensive and low throughput of animal models. A crucial component in this development is the availability of practical and efficient computational methods, which we have shown to be achievable with a combination of both novel and existing image analytic practices and robust statistical analysis. A prerequisite for expanding the scope of PDX cell culture-based drug testing *in vitro*, is thorough experimental validation of mouse versus cell culture models. For our NSCLC PDX cultures, the obtained drug responses showed a similar trend in *in vitro* and *in vivo* (Figs 6 and 9). Most importantly, the scope or total number of compounds that can be tested in PDX models can be significantly expanded by complementing such tests with matching organotypic PDX 3D cultures. These can serve as a valid, relatively inexpensive tool for excluding compounds that will likely prove ineffective *in vivo*. Therefore, drug doses likely to be effective in animals could be pre-screened *in vitro*, and potential toxic side effects might be recognized and avoided. Taken together, our newly developed biological and computational methods open up novel opportunities for high-content drug screening based on patient-derived cell cultures, and personalized chemosensitivity testing.

## Electronic supplementary material

Supplementary Information
