# Supplementary material for: A high-content image analysis approach for quantitative measurements of chemosensitivity in patient-derived tumor microtissues
Source: Sci Rep. 2017 Jul 26;7:6600. doi: 10.1038/s41598-017-06544-x (PMC5529420; doi:10.1038/s41598-017-06544-x)
Supplement: Supplementary file 1 — Supplementary Information [file 41598_2017_6544_MOESM1_ESM.pdf]

Supplemental material to: A high-content image analysis approach for quantitative measurements of chemosensitivity in patient-derived tumor microtissues

Ilmari Ahonen[1,2,+,*], Malin Akerfelt[2,+], Mervi Toriseva[2], Eva Oswald[3], Julia Schüler[3], and Matthias Nees[2]

[1]Department of Mathematics and Statistics, University of Turku, Finland
[2]Institute of Biomedicine, University of Turku, Finland
[3]Discovery Services; Charles River, Freiburg, Germany
[*]ilmari.ahonen@utu.fi
[+]these authors contributed equally to this work

June 1, 2017

Supplemental methods

Thinning algorithm

Thinning of the image mask is obtained with a sequence of hit-and-miss transformations with a specific set of patterns. Consider an $r \times c$ image mask M, where each pixel $m_{ij}, i = 1, \ldots, r, j = 1, \ldots, c$ has a value 0 when it belongs to the background and value 1, when it belongs to the foreground. A hit-and-miss transformation with a 3×3 pattern matrix P is performed by sliding P over all possible positions in M and comparing the values in P against those in M. If the values in P match those in the given position of M, that position's center pixel is set to the foreground. Otherwise, the pixel is set to the background. Thinning is achieved by applying the hit-and-miss transformation subsequently with the following two patterns and all of their four rotations:

$$\begin{pmatrix} 0 & 0 & 0 \\ & 1 & \\ 1 & 1 & 1 \end{pmatrix}, \quad \begin{pmatrix} & 0 & 0 \\ 1 & 1 & 0 \\ & 1 & \end{pmatrix}$$

The empty cells in the patterns are treated as wild cards and they do not affect the comparison.

In the paper, two iterations of this thinning algorithm are applied, resulting in a total of 16 hit-and-miss transformations, where both patterns and their rotations are used twice.

PDX tumor sequencing and gene expression analyses

Total RNA sample was digested by DnaseI (NEB), purified by oligo-dT beads (Dynabeads mRNA purification kit, Invitrogen), then poly(A)-containing mRNA were fragmented into 200-250bp with Fragment buffer (Ambion). Double strand cDNA synthesis and (standard or strand-specific) sequencing libraries were prepared and validated following the sequencing provider's RNA-Seq protocols. Sequencing was done using Illumina HiSeq-2000/2500/4000 in 100/126bp paired-end (PE) reads with an expected throughput of 10G bases per sample.

PE reads were independently mapped to the Human hg19 and the Mouse mm10 reference genome with Tophat2 (splice junction mapper for RNA-Seq reads using the Bowtie aligner) by providing gene and transcript annotations allowing a pre-mapping of reads to the transcriptome. To remove the mouse stroma content, PE reads that mapped better on the mouse than on the human genome were discarded from the Human mapped read dataset (based on the Tophat mapping score) using PicardTools. Human specific mapped reads were then processed by the Cufflinks RNA-Seq workflow to get normalized expression tables: generation of assembled transcripts with Cufflinks, gene and transcript quantification with Cuffquant and normalization in FPKM units with Cuffnorm. A final matrix of gene or isoform expression based on FPKM values was created.

Raw reads were subjected to fastQC to calculate read quality metrics. After the alignment to the Human reference genome and Mouse reads removal, the quality of BAM files was assessed by RNA-SeQC to obtain the percentage of transcripts-associated reads and mean coverage metrics for low, medium or high expressed transcripts.

The versions and parameters of the software used in the Oncotest V1.1 RNASEQ analysis pipeline for RNA-sequencing are listed in the Supplemental Table 2, and the utilized annotation databases in Supplemental Table 3.

Supplemental figures

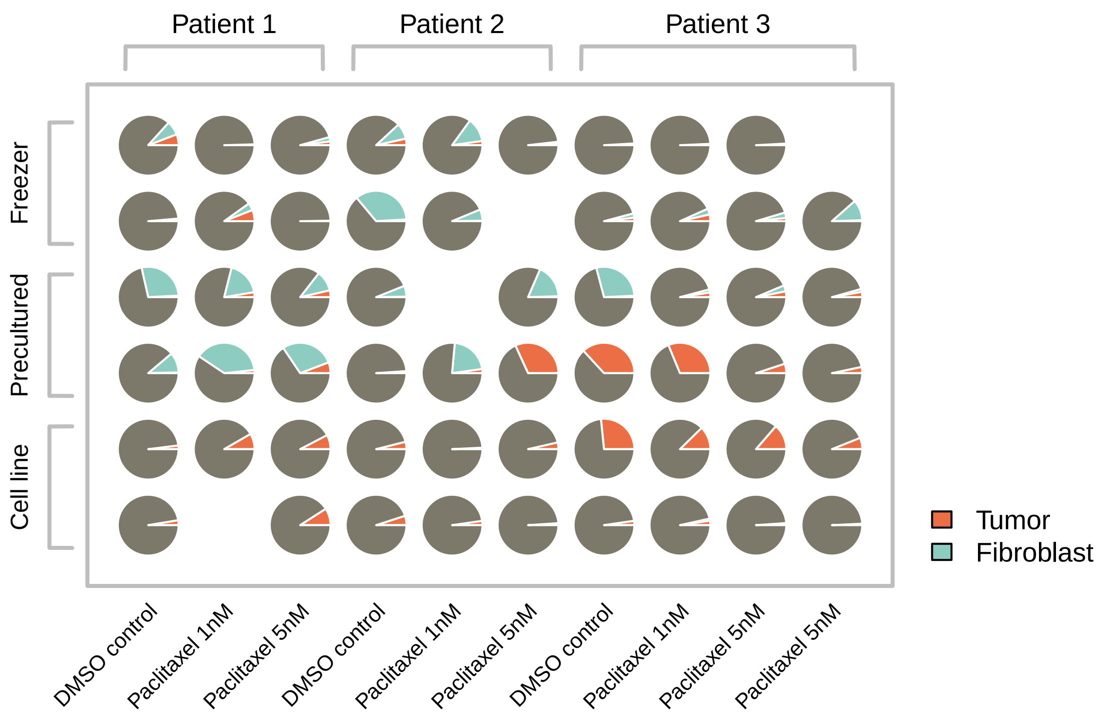

Figure 1: ***Design of the first PDX data set*** *The pie charts display the estimated total area covered by each tissue type.*

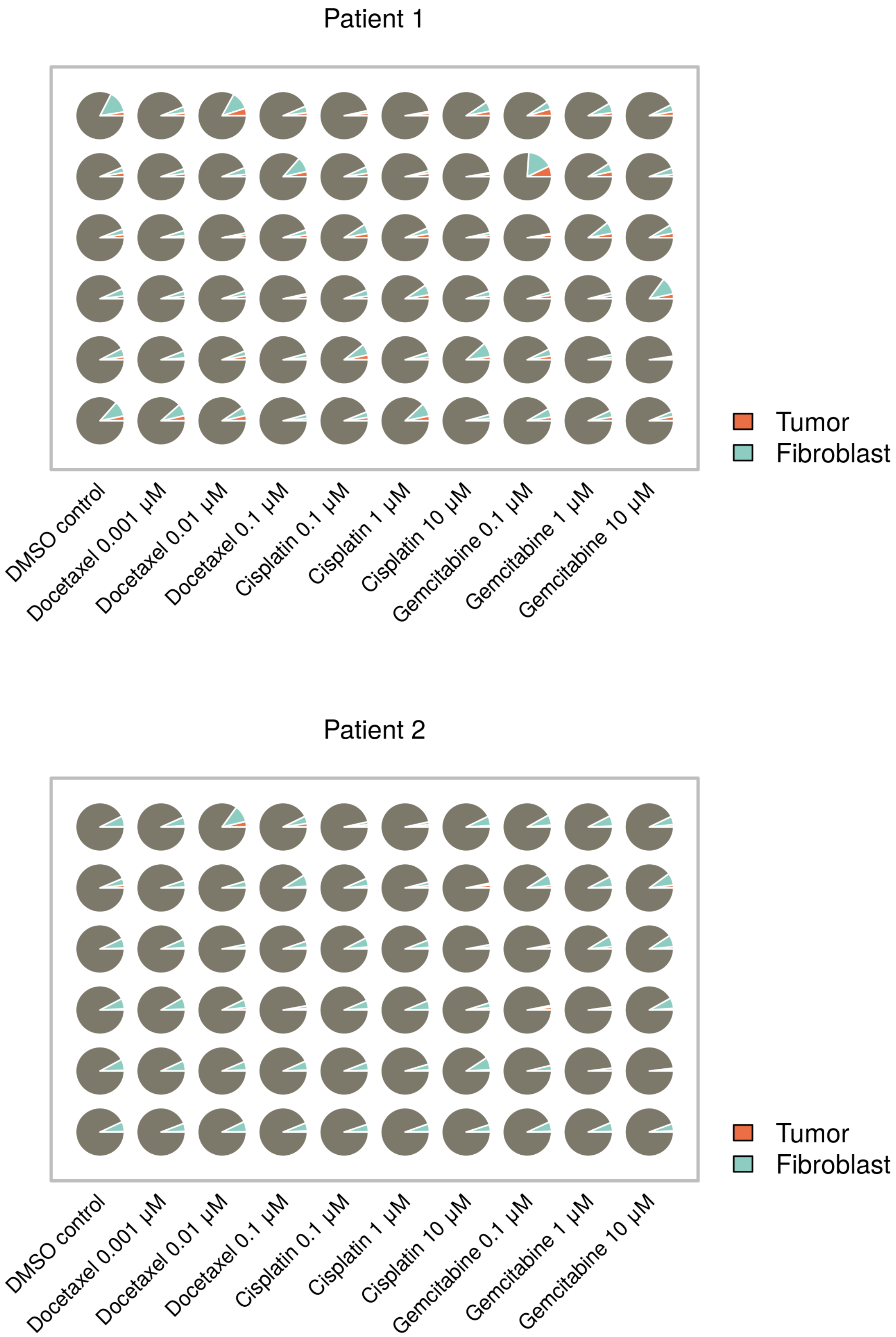

Figure 2: ***Design of the second PDX data set*** *The pie charts display the estimated total area covered by each tissue type.*

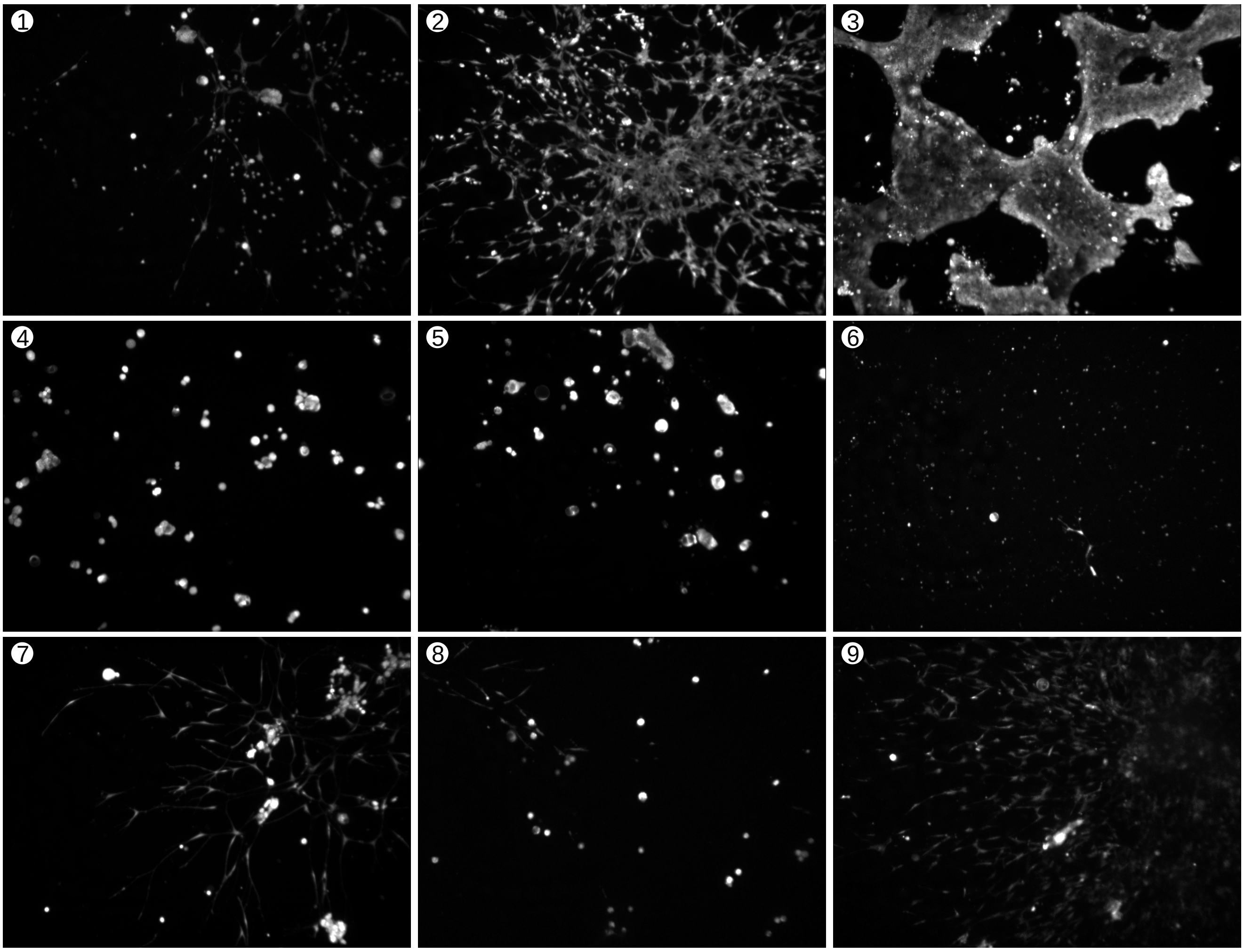

Figure 3: ***The original ground truth images***

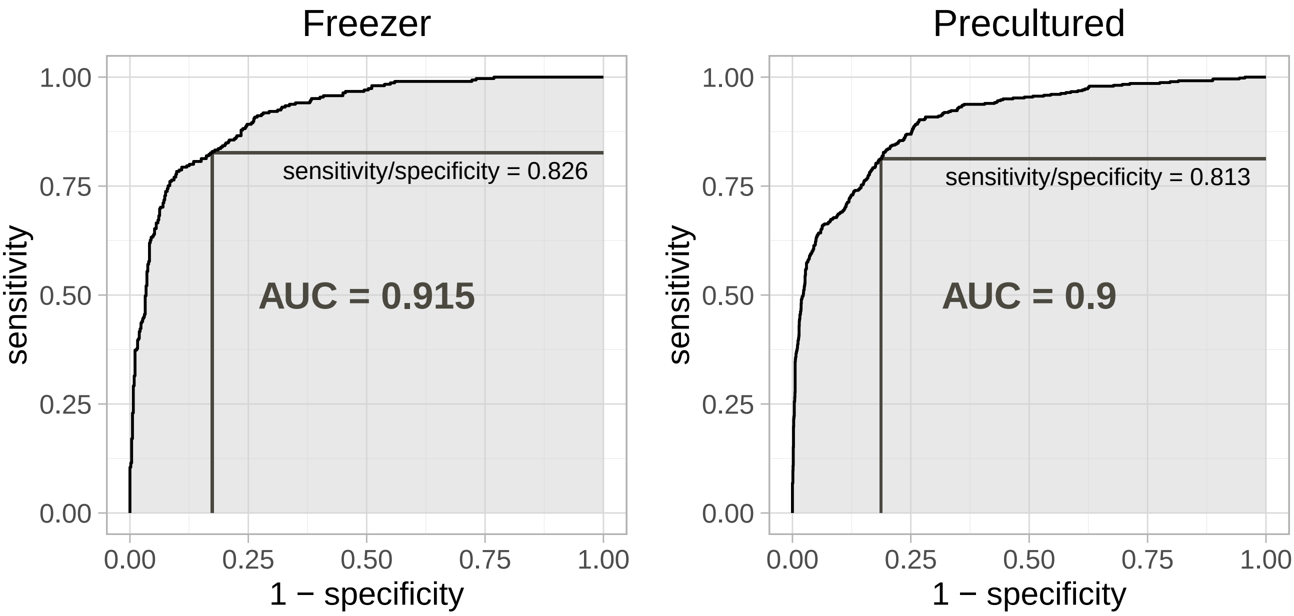

Figure 4: ***The performance of the chosen classifier in the complete training data illustrated with ROC-curves***

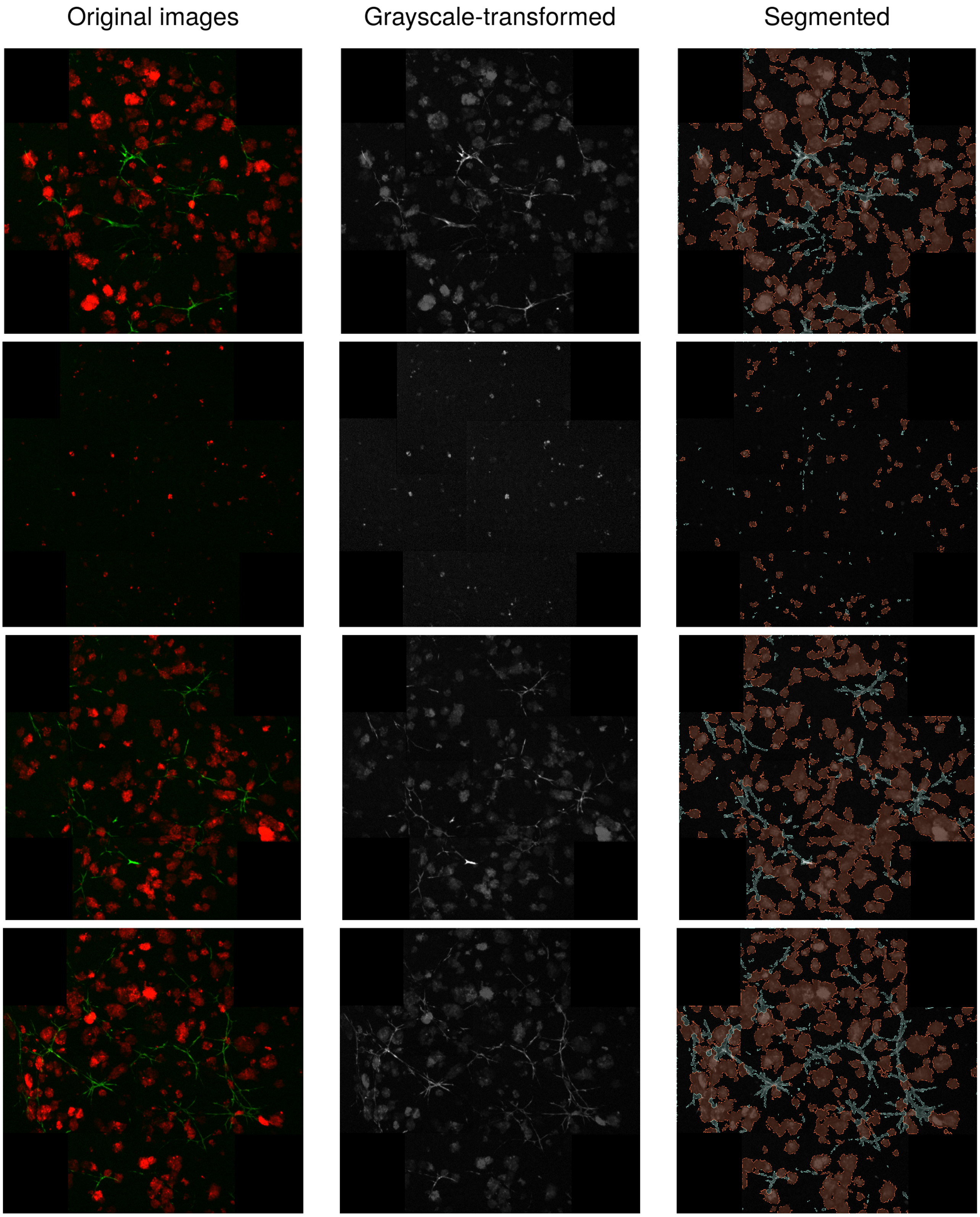

Figure 5: ***Examples of segmented cell line co-culture data.***

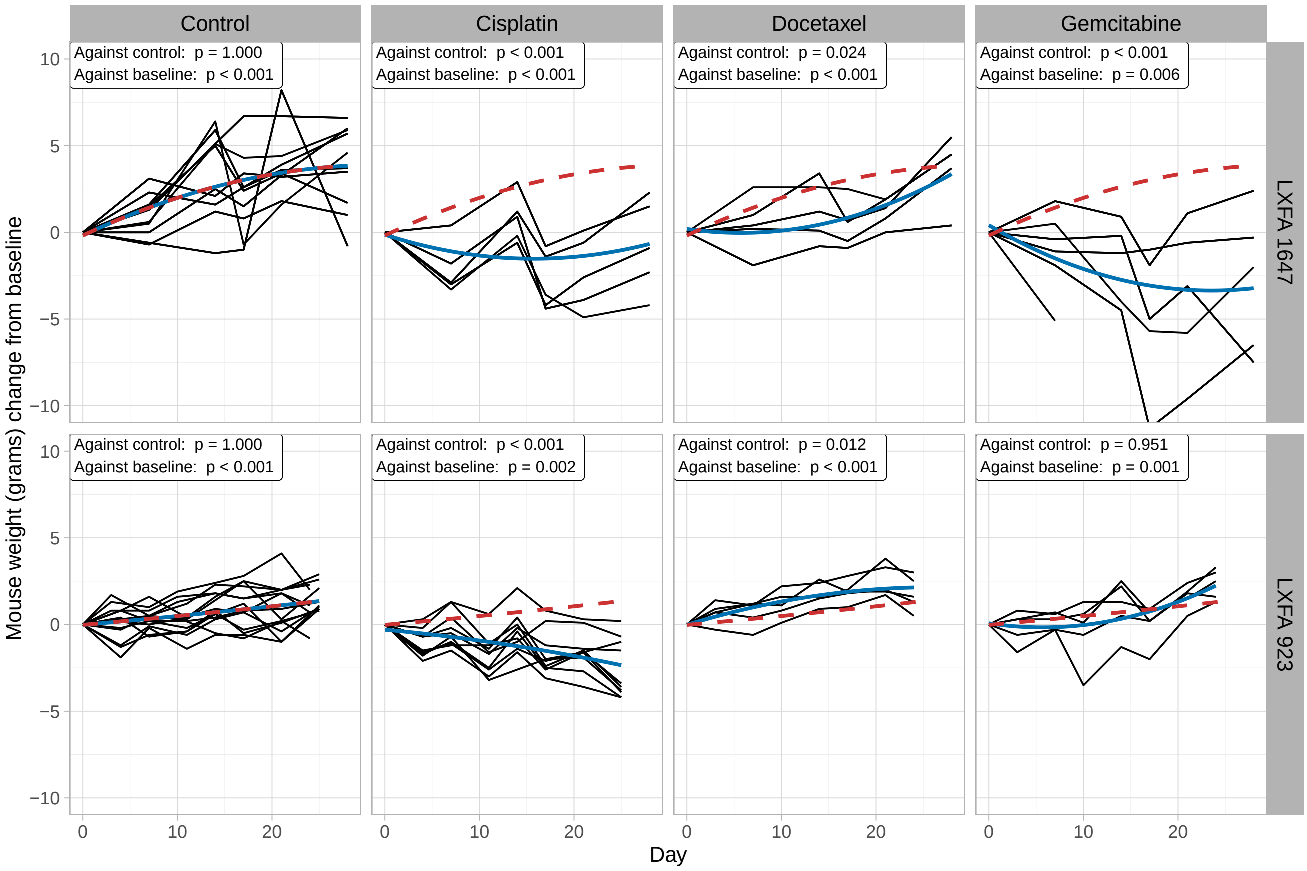

Figure 6: ***Drug toxicity in mouse xenograft experiments.*** *Each black line represents repeated measurements of mouse weight, blue solid line the treatment average and red dashed line the control average. The average fits are obtained with a simple second degree polynomial. Two statistical tests are performed: one against the control treatment and one against the baseline. Experiments with Docetaxel and Gemcitabine were not conducted with patient LXFA 923.*

Supplemental tables

Tumor Designation	Mutation Analyses					Protein Analyses			
	TP53	Kras	EGFR	MET	lkb1	P-Met	HGF (pg/ml)	P-EGFR	t-EGFR
LXFA 923	wt	KRAS: G12C; heterozygous	wt	wt	LKB1: c. 842insC, p. L282fsX2; homozygous	0		2.15	0.32
LXFA 983	Y163C; homocygous	KRAS: G12C; heterozygous	wt	wt	wt	0	-	0.74	0.17
LXFA 1647	L265P; homocygous	wt	wt	wt	wt	3	0	0.22	0.06

Table 1: ***Mutations of the PDX samples***

Tool	Version	Command line parameters if different from the default
fastQC	0.10.1	
Tophat	2.0.13	-no-mixed -library-type fr-unstranded (or fr-firststrand) -GTF genes.gtf -transcriptome-index=trancriptome
Bowtie	1.1.1	
Bowtie2	2.2.1	
STAR	2.4.0	
PicardTools	1.96	
SamTools	0.1.19	
Cufflinks	2.2.1	
Qualimap	2.1	
RNA-SeQC	1.1.8	-n 1000 -t gencode.v7.annotation.gtf -r -strat gc -gc gencode.v7.gc.txt bwa -BWArRNA human_all_rRNA.fasta

Table 2: ***The versions and parameters of the software used in the Oncotest V1.1 RNASEQ analysis pipeline for RNA-sequencing.***

Database	Version	Availability
Human genome	hg19	`ftp://ussd-ftp.illumina.com/Homo_sapiens/UCSC/hg19/`
Mouse genome	mm10	`ftp://ussd-ftp.illumina.com/Mus_musculus/UCSC/mm10/`
GENCODE	v7	`http://www.gencodegenes.org/releases/7.html`

Table 3: ***The versions and sources of the annotation databases used in RNA-sequencing.***
